# Triple-Step Laparoscopic Incisional Hernia Repair: Midline Suture Closure Supported by Dorsal Component Separation and Intraperitoneal Onlay Mesh Reinforcement

**DOI:** 10.1007/s00268-014-2747-0

**Published:** 2014-09-05

**Authors:** Christoph W. Strey

**Affiliations:** Department of General, Visceral and Vascular Surgery, Diakoniekrankenhaus Friederikenstift, 5 Humboldtstrasse, 30169 Hannover, Germany

## Abstract

Incisional hernias remain a surgical challenge when balancing surgical morbidity, functional restoration, and risk of recurrence. Laparoscopic intraperitoneal onlay mesh (IPOM) placement reduces postoperative wound infections and allows fast patient recovery. Yet, current IPOM techniques do not achieve closure of the midline hernia gap, thereby increasing the risk of persistent mesh bulging with poor abdominal wall function. We propose a novel triple-step hernia repair technique that includes tension-free midline reconstruction. It is achieved through laparoscopic dorsal component separation and laparoscopic suture closure of the midline with a 1.0 polydioxanone suture sling. Combining dorsal abdominal wall component separation, a midline closure with adequate suture strength, and IPOM reinforcement merges the benefits of open and laparoscopic hernia repair. This triple-step technique allows static and functional laparoscopic abdominal wall reconstruction.

**Electronic supplementary material:**

The online version of this article (doi:10.1007/s00268-014-2747-0) contains supplementary material, which is available to authorized users.

## Introduction

Laparoscopic intraperitoneal onlay mesh placement with omission of laparotomy for hernia repair is beneficial for patients’ physical function and reduces the incidence of surgical-site infections. Functional restoration of the abdominal wall is more effective, however, with open hernia repair, which allows reconstruction of the midline with approximation of the rectus abdominis muscle. Another disadvantage of not closing the hernia gap during laparoscopic intraperitoneal onlay mesh (IPOM) placement is the subsequent development of seroma of the original hernia sac or a bulging phenomenon caused by herniation of the mesh through the gap. We propose a triple-step procedure to compensate for some of the shortcomings of simple laparoscopic IPOM placement: (1) laparoscopic transversus abdominis fascia release; (2) laparoscopic use of a regular slowly absorbable polydioxanone suture (PDS) sling (1.0) for fascial closure; (3) IPOM reinforcement of the closed hernia gap.

## Methods

The patient is placed in supine position with both arms alongside the abdomen. The surgeon and first assistant stand on the right side of the patient with the monitor opposite on the left side. The scrub nurse stands to the right of the surgeons. A mini-laparotomy is made close to the right ventral axillary line in mid-abdomen. After capnoperitoneum is established via a 10-mm trocar, two additional 5-mm trocars are inserted close to the rib cage and in the lower right abdomen, respectively. Initially, intraabdominal pressure is adjusted to 12 mmHg. Adhesions to the abdominal wall must be taken down, including removal of relevant preperitoneal fat to delineate the abdominal fascia. Similarly, the fascial wound edge is cleared from adherent tissue to enable well-defined stitch placement. By applying external pressure, the hernia sac can be inverted into the abdomen to facilitate its laparoscopic removal by carefully avoiding injury to the adjacent skin. All resected tissues can be extracted using an endobag. Afterward, laparoscopic dorsal component separation is performed as described by Milburn et al. [1]. With this first step of the proposed triple-step procedure, the transversus abdominis fascia is detached from its medial fixation at the border of the rectus sheath (video 1). In contrast to the open dorsal procedure described by Novitsky et al. [2], the rectus sheath and the transverse abdominis muscle remain intact, and the perforator vessels and nerves are less apt to be injured.

**Fig. 1:**
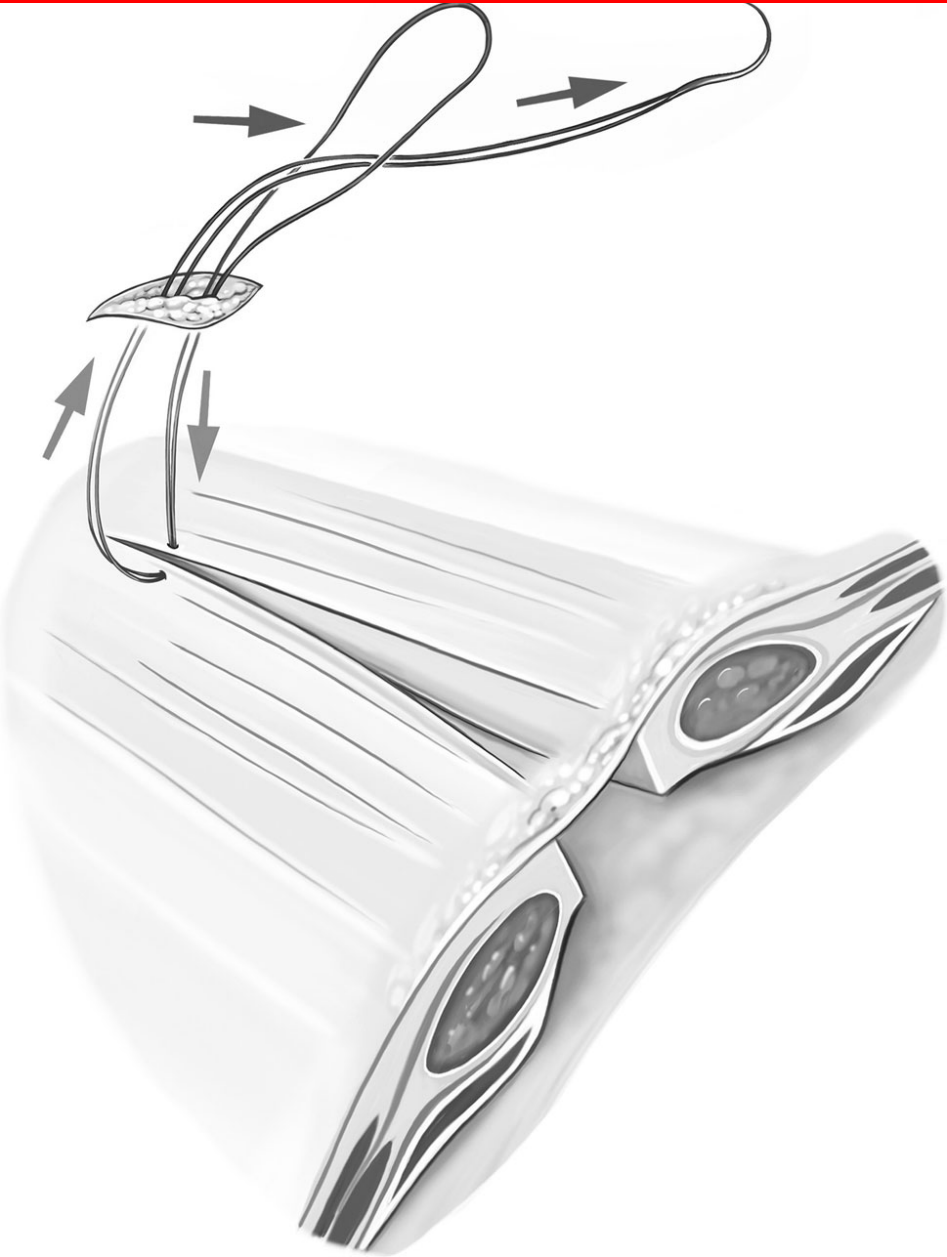
Polydioxanone suture (PDS) sling is secured trans-subcutaneously through a 5-mm skin incision cephalad of the fascial gap

**Fig. 2:**
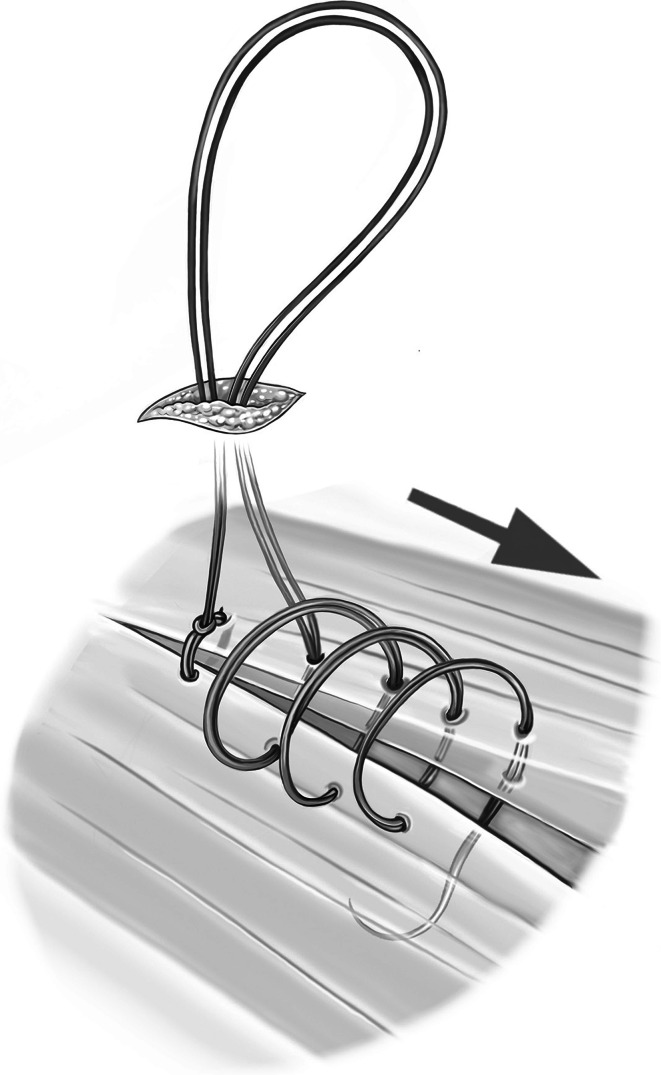
External view of the progress of the laparoscopically performed suture line. The PDS sling is subsequently pulled into the abdomen as additional length is required

**Fig. 3:**
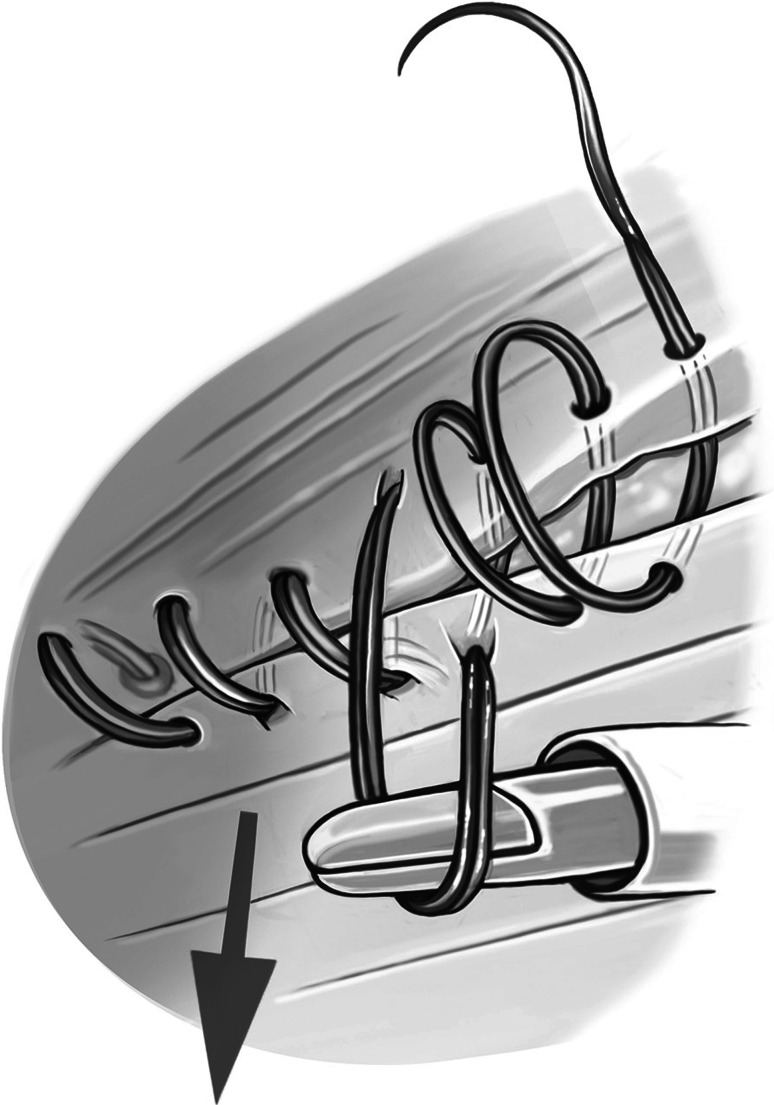
Intraabdominal view of the suture line as the PDS sling is advanced and tightened using a blunt laparoscopic instrument in a manner comparable to tying shoelaces

**Fig. 4:**
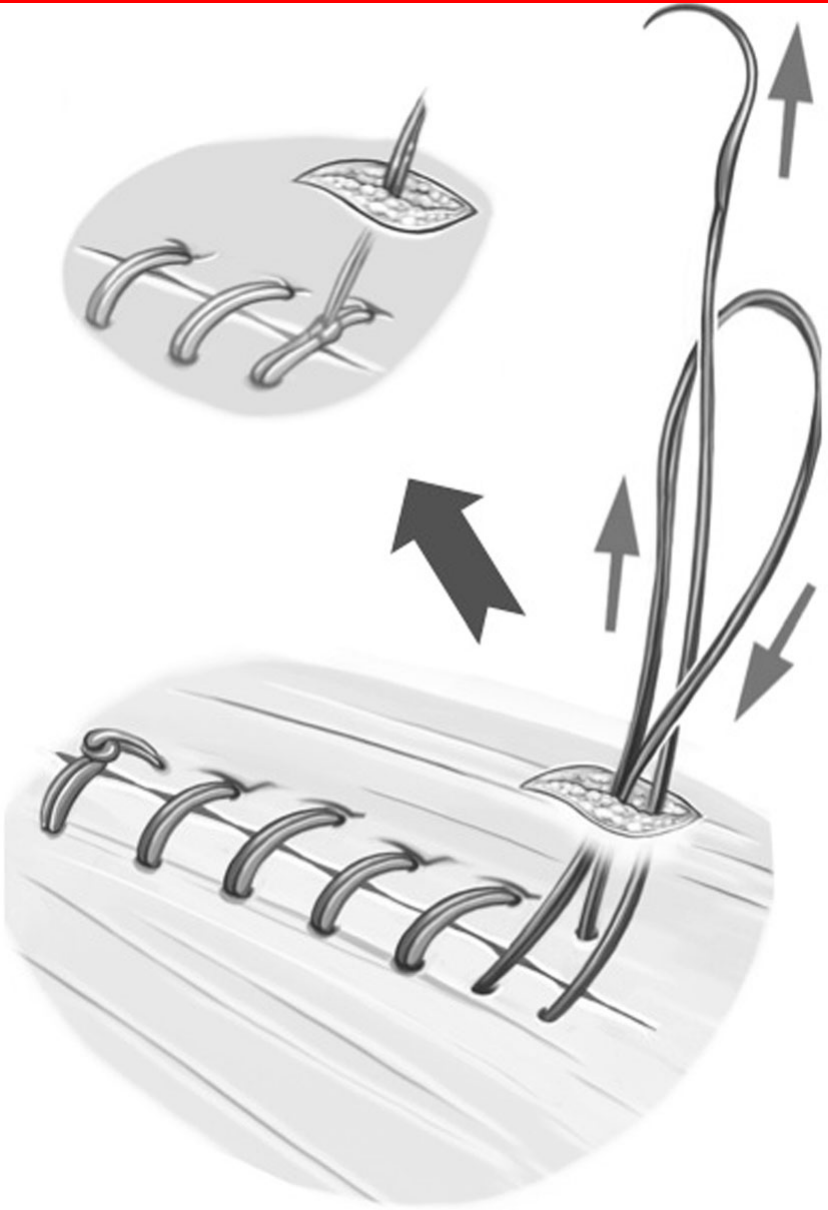
After completion of the suture line, the PDS sling is passed through the abdominal wall to the exterior and tied with an Aberdeen knot

The second step of the operation is now initiated. The needle of the PDS sling is inserted cephalad from the hernia gap via a 5-mm skin incision. The needle is then reversed under laparoscopic control and extracted via the same skin incision (Fig. 1). The sling is engaged, and the needle is again inserted into the abdomen while the major part of the sling suture remains external (video 2). This facilitates laparoscopic handling of the sling, and the whole length of the sling can be preserved. The sling can now be stitched laparoscopically, carefully controlling the stitch width (Fig. 2). The comparatively large size of the needle allows adequate stitch length. The monofilament suture material must not be damaged so as to avoid the risk of disconnecting the needle. As the suture line proceeds, the required suture length can be provided from the external part of the sling. With this technique, the amount of suture in the abdomen can be kept to a minimum to facilitate laparoscopic handling while suturing. After completion of the suture line, the needle is extracted through an additional incision at the foot end of the hernia gap. The suture can now be pulled through manually with laparoscopic assistance in a manner comparable to tying shoelaces (video 2; Fig. 3). Before applying the final tension of the suture, abdominal pressure must be reduced to 6 mmHg to facilitate approximation of the fascial edges. After that is accomplished, the needle is once again inserted into the abdomen, reversed, and extracted, thereby generating an external loop. Final tying is done with an Aberdeen knot [3] (Fig. 4). The Aberdeen knot allows the needle to remain connected to the suture, which helps bury its end in the subcutaneous tissue after securing the knot.

Laparoscopic IPOM placement as described elsewhere [4] is the third step that completes the operation. Sufficient mesh overlap can be established more easily after the hernia gap has been closed.

So far, 11 patients (4 male, 7 female) have undergone laparoscopic closure of the fascial gap prior to IPOM reinforcement. Among them, three patients required laparoscopic dorsal component separation to reduce fascial tension. No surgical-site infections or signs of recurrence have been noted. There have been no seromas or signs of bulging. In all cases, the abdominal shape was restored with closure of the fascial gap.

## Conclusions

We propose a new, simple laparoscopic triple-step technique that meets the requirements of incisional hernia repair concerning abdominal wall reinforcement and tension-free repair. The midline closure is established using a strong PDS sling suture regularly applied during open surgery together with dorsal component separation that consists of transversus abdominis fascia release. Final IPOM reinforcement ensures the strength of the hernia repair. Our technique also follows the recommendations for abdominal fascial closure in terms of the stitch length and the suture length/wound length ratio of at least 4 [5]. We find the procedure to be safe and reliable without relevant additional morbidity risk for the patient. We believe that the procedure can become a valuable asset to the surgical armamentarium in the treatment of incisional hernias by combining the benefits of laparoscopic and open hernia repair.

## Electronic supplementary material

Below is the link to the electronic supplementary material.

Supplementary material 1 (M4 V 19700 kb)

Supplementary material 2 (M4 V 15733 kb)
